# A Fusion–Growth Protocell Model Based on Vesicle Interactions with Pyrite Particles

**DOI:** 10.3390/molecules29112664

**Published:** 2024-06-04

**Authors:** Dong Guo, Ziyue Zhang, Jichao Sun, Hui Zhao, Wanguo Hou, Na Du

**Affiliations:** 1 Key Laboratory of Colloid and Interface Chemistry (Ministry of Education), School of Chemistry and Chemical Engineering, Shandong University, Jinan 250100, China; 2 National Engineering Technology Research Center for Colloidal Materials, Shandong University, Jinan 250100, China

**Keywords:** vesicle, single-chain amphiphiles, pyrite, solid–liquid interface, protocell

## Abstract

Protocell models play a pivotal role in the exploration of the origin of life. Vesicles are one type of protocell model that have attracted much attention. Simple single-chain amphiphiles (SACs) and organic small molecules (OSMs) possess primitive relevance and were most likely the building blocks of protocells on the early Earth. OSM@SAC vesicles have been considered to be plausible protocell models. Pyrite (FeS_2_), a mineral with primitive relevance, is ubiquitous in nature and plays a crucial role in the exploration of the origin of life in the mineral–water interface scenario. “How do protocell models based on OSM@SAC vesicles interact with a mineral–water interface scenario that simulates a primitive Earth environment” remains an unresolved question. Hence, we select primitive relevant sodium monododecyl phosphate (SDP), isopentenol (IPN) and pyrite (FeS_2_) mineral particles to build a protocell model. The model investigates the basic physical and chemical properties of FeS_2_ particles and reveals the effects of the size, content and duration of interaction of FeS_2_ particles on IPN@SDP vesicles. This deepens the understanding of protocell growth mechanisms in scenarios of mineral–water interfaces in primitive Earth environments and provides new information for the exploration of the origin of life.

## 1. Introduction

“How does life originate? How can non-living inorganic matter transform into organic life forms through complex systems? Do humans have the ability to create life from scratch? Can we control complex systems like cells?” were identified as some of the world’s 125 most cutting-edge major scientific questions, as published in the journal *Science* in 2021 [1]. The origin of cellular life remains elusive, with no definitive answer. Primitive life depends on the self-organizing properties of its constituent parts, as well as on the input of energy and matter from the environment, to execute the most fundamental cellular processes [2]. Protocells, regarded as the earliest life-like entities, are hypothesized to consist of three essential components: membrane-forming molecules, information molecules and catalytic molecules [3]. In the origin of life, energy supply was imperative. In 1988, Wächtershäuser postulated the hypothesis of the origin of life within an iron–sulfur environment [4,5], arguing that at the origin of life, energy supply was primitive autotrophic metabolism [6]. The energy sources hypothesized to have driven the origin of life included iron sulfides and other minerals (e.g., pyrite). The energy released from the redox reactions of these metal sulfides could have facilitated the synthesis not only of organic molecules but also of oligomers or polymers. These systems could have developed sets of autocatalytic systems capable of self-replication, as well as living and independent entities capable of metabolizing life forms prior to those known to us today [4]. In contemporary geochemical environments, the population of prokaryotic cells within biofilm communities residing at mineral–water interfaces surpasses by orders of magnitude those inhabiting water environments, owing to the manifold metabolic and protective functions these interfaces offer [7,8,9]. Hence, it is a plausible conjecture that protocells on the primordial Earth were in proximity to mineral–water interfaces [10,11,12,13].

Through the compositional analysis of meteorite extracts [14,15] and experiments simulating primordial Earth environmental conditions [10,11], researchers have identified a range of organic molecules pertinent to early Earth conditions: (1) lipid-like compounds capable of forming protocell structures, consisting of simple single-chained amphiphilic molecules (SCAs) such as fatty acids, alkyl keto acids and monoalkyl phosphates [16,17,18,19,20,21,22]; (2) organic small molecules (OSMs) containing L-amino acids, purines, pyrimidines, D-ribose and isoprenoid derivatives, etc. [23,24,25]. It can be deduced that these SCAs and OSMs, demonstrated to possess primitive relevance, were probably constituents of protocells during the early stages of Earth. SCAs have been extensively employed as modeling units for protocell membranes in studies on the origin of life [26,27,28,29] It has been shown that specific minerals can play some key roles as catalysts [30,31,32,33]. For instance, they have been shown to catalyze the formation of RNA polymers [34] and peptides [35], while minerals can enhance the initial assembly rate of amphiphiles into vesicles, ultimately contributing to the formation of protocell membranes [36,37]. The iron group elements Fe, Co and Ni serve as the most effective and versatile catalysts of life, and Fe is the most prominent and has the greatest geochemical abundance. The most stable compounds of the iron family elements and the most prominent iron compounds under anaerobic conditions are sulfides [38]. The transition from inanimate to living matter may result from the self-assembling properties of organic molecules and their interaction with the chemical diverse inorganic environment [2]. So, what kind of “sparks” can collide between minerals of primordial relevance and the “OSM@SCA vesicle” protocell model?

In this study, we select primitive relevant sodium monododecyl phosphate (SDP), isopentenol (IPN) and pyrite (FeS_2_) mineral particles to establish a protocell model. We investigate the fundamental physical and chemical properties of FeS_2_ particles and reveal the effects of the FeS_2_ size, content and duration of interaction on IPN@SDP vesicles. Through simulating scenarios of the mineral–water interface in the primitive Earth environment, the changing rules of morphology, size and structure of the protocell model system are explored to provide information for the exploration of the origin of life.

## 2. Results and Discussion

### 2.1. Basic Physical and Chemical Properties of FeS_2_

**Figure 1:**
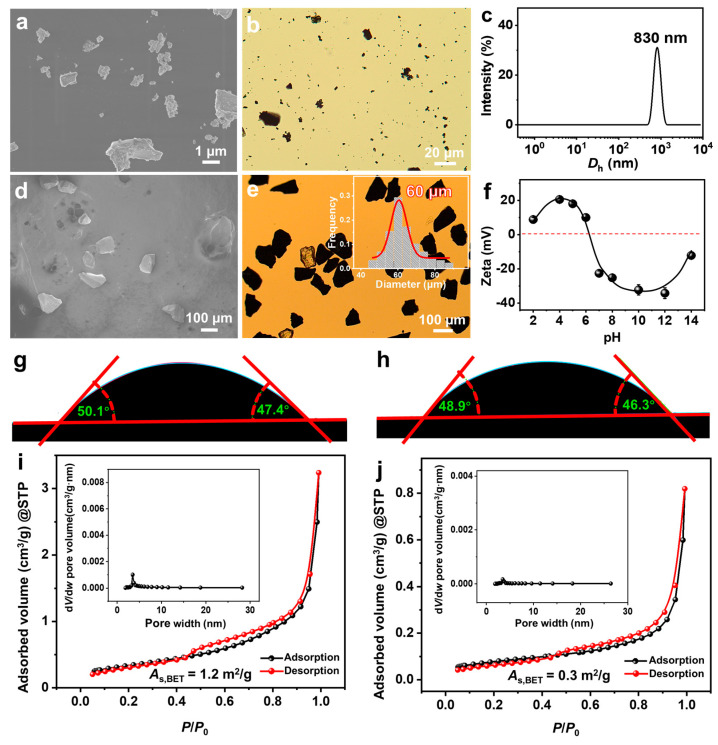
(**a**,**d**) SEM images, (**b**,**e**) OM images, (**c**) DLS size distributions, (**f**) zeta potential versus pH curves (25.0 ± 0.5 °C), (**g**,**h**) contact angles of water on the surface of FeS_2_ particles and (**i**,**j**) nitrogen adsorption–desorption isotherms for (**a**–**c**,**f**,**g**,**i**) FeS_2_-1 and (**d**,**e**,**h**,**j**) FeS_2_-60, with the inset in e being a histogram of the size distribution of FeS_2_-60, and the insets in (**i**,**j**) being the pore size distributions of BJH.

From scanning electron microscope (SEM) (Figure 1a) and optical microscope (OM) (Figure 1b) observations, FeS_2_-1 is an irregularly shaped massive particle with a size of ~1 μm. The peak of its dynamic light scattering (DLS) size distribution is located at ~830 nm (Figure 1c), which is consistent with the results of the electron microscopy observations. FeS_2_-60 is an irregularly shaped massive particle (Figure 1d,e) with a size of 60 ± 15 μm. The isoelectric point of FeS_2_ particles in water is ~6.6 (Figure 1f), which is consistent with the values reported in the literature [39]. The p*K*_a_ (where *K*_a_ is the acid dissociation constant) values of SDP are 2.85 (p*K*_a1_) and 7.35 (p*K*_a2_) [40]. The pH of the SDP/IPN/H_2_O homogeneous solution is measured as 6.30–6.50, close to p*K*_a2_ (7.35). This indicates that under the studied conditions, the main forms of SDP are C_12_H_25_OP(OH)O_2_Na and C_12_H_25_OPO_3_Na_2_, with trace amounts of C_12_H_25_OP(OH)_2_. Under this circumstance, those FeS_2_ particles should be slightly positively charged. The contact angles of water on the surfaces of FeS_2_-1 and FeS_2_-60 particles are 48 ± 3° and 47 ± 3°, respectively (Figure 1g,h), indicating that surfaces of FeS_2_ mineral particles are hydrophilic. Type IV adsorption isotherms and H3-type hysteresis loops are obtained through nitrogen adsorption and desorption measurements (Figure 1i,j). Combining the results of the Barrett–Joyner–Halenda (BJH) pore-size distributions, it is revealed that the FeS_2_ particles construct few slit mesopores by particle stacking. The Brunauer–Emmett–Teller (BET) specific surface areas of FeS_2_-1 and FeS_2_-60 are 1.2 ± 0.1 m^2^/g and 0.3 ± 0.1 m^2^/g, respectively, with the former being ~4 times that of the latter.

### 2.2. Effect of FeS_2_ Particles on Vesicles

**Figure 2:**
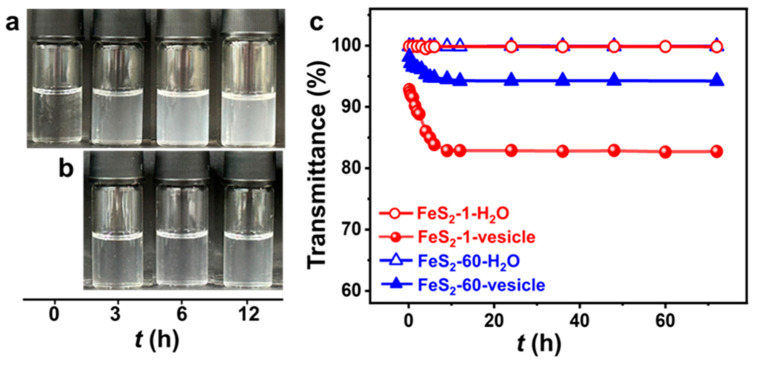
(**a**,**b**) Photographs of the appearance and (**c**) transmittance curves (25.0 ± 0.5 °C) of FeS_2_-vesicle solutions at different times. Transmittance is the average of three measurements. (**a**) FeS_2_-1-vesicle, (**b**) FeS_2_-60-vesicle.

The vesicle phase is detected in the SDP/IPN/H_2_O ternary system in the water-rich zone (Figure S1). The concentrations of SDP and IPN are quite low, and the appearance of the vesicle solution is clear and transparent (Figure 2a). The appearance of the FeS_2_-vesicle solutions turns turbid upon the interaction time increasing (Figure 2a,b). The transmittance of the FeS_2_-vesicle solution decreases gradually from ~99% to ~83% and ~94% with the addition of FeS_2_-1 and FeS_2_-60 (Figure 2c), respectively, and reaches a constant in ~12 h. In contrast, the supernatants of FeS_2_-H_2_O show no change at all for the transmittance test. FeS_2_ particles with smaller size enhance the turbidity of the FeS_2_-vesicle solution more significantly than the case of FeS_2_-60, which may be attributed to its higher specific surface area (Figure 1i,j).

**Figure 3:**
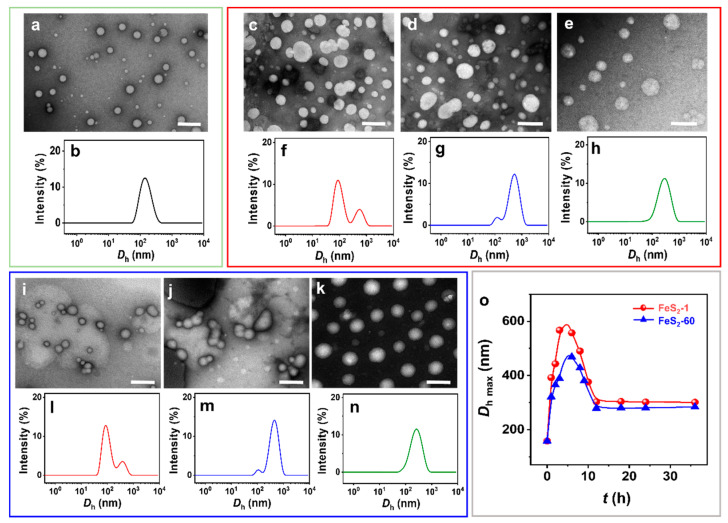
(**a**,**c**–**e**,**i**–**k**) NS-TEM images and (**b**,**f**–**h**,**l**–**n**) DLS size distributions of (**a**,**b**) vesicle, (**c**–**h**) FeS_2_-1-vesicle and (**i**–**n**) FeS_2_-60-vesicle solutions at different times. (**o**) DLS size distribution of FeS_2_-vesicle solutions as a function of time. (**c**,**f**,**i**,**l**) 3 h; (**d**,**g**,**j**,**m**) 6 h; (**e**,**h**,**k**,**n**) 12 h. Scale bar: 500 nm.

The spherical IPN@SDP vesicles are observed by negative staining transmission electron microscopy (NS-TEM) (Figure 3a). After the addition of FeS_2_, the vesicles vary gradually from spherical to dumbbell-shaped, then to ellipsoidal, and finally to spherical vesicles again, but with larger sizes (Figure 3c–e,i–k). According to the results of size distribution, the vesicle size distribution peak is initially located at ~150 nm (Figure 3b). Upon the interaction between FeS_2_ particles and vesicles, a new peak appears at 500–600 nm (Figure 3f,l), probably representing the size peak of the dumbbell-shaped vesicles. Then, the peak area of the new peak gradually increases, and the peak of the size distribution is located at ~400 nm. This may result from the fusion of the two spheres in the dumbbell-shaped vesicles, namely ellipsoidal vesicles (Figure 3g,m). Ultimately, the size distribution peak is located at ~300 nm, corresponding to the spherical vesicles with larger sizes than the vesicles without FeS_2_ particles (Figure 3h,n). The DLS results generally agree with the NS-TEM observations. In summary, the FeS_2_ particles induce the morphology transition of sphere–dumbbell–ellipsoid–larger sphere vesicles, which may be the intrinsic reason for the increase in the turbidity of the vesicular solution under the effect of FeS_2_ particles. This transition takes 12 h to establish a dynamic equilibrium state, which is also consistent with the results of turbidity tests (Figure 2). Compared to the original pyrite samples, the water contact angles (*θ*_w_) of the FeS_2_ mixed with vesicle solution are decreased from 47–48° to 26–27° (Figure S2a,b), respectively. The decrease in *θ*_w_ is probably attributed to the adsorption of the amphiphilic bilayer [41,42,43] on the particle surface. Moreover, the *θ*_w_ values of FeS_2_ samples that interact with the vesicle solutions increase to 46–48° when they are subjected to ultrasonic treatment in water (Figure S2c,d), which are almost the same values as the original FeS_2_ particle samples (Figure 1g,h). Therefore, it is reasonable to believe that the desorption of SDP or IPN molecules on the FeS_2_–water interface could be induced and accelerated by certain energy inputs (e.g., shaking, stirring and ultrasonic agitation) [44,45]. The dynamics of the adsorption-desorption equilibrium on the water–FeS_2_ interface probably play an important role in this required time (12 h) to establish the apparent equilibrium states of the FeS_2_-vesicle samples.

Further, the increase in vesicle size after interaction with FeS_2_-1 is more pronounced than the one with FeS_2_-60 (Figure 3o), presumably due to the larger specific surface area of FeS_2_-1. In addition, there is no significant change in the appearance, morphology and size of IPN@SDP vesicles after storage at 25.0 ± 5.0 °C for 6 months (Figure S3), which is similar with those solid interface-induced, simple single-chained amphiphilic molecule (SCA) vesicles in our previous study [12,42].

### 2.3. Influence of FeS_2_ Particle Content

**Figure 4:**
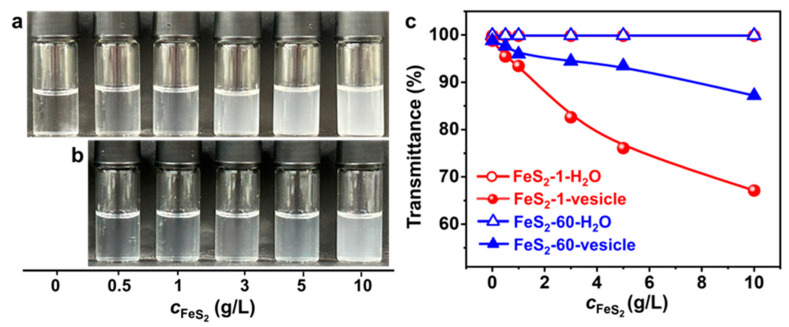
(**a**,**b**) Photographs of the appearance and (**c**) transmittance of FeS_2_-vesicle solutions at different particle contents (24 h). Transmittance is the average of three measurements. (**a**) FeS_2_-1-vesicle, (**b**) FeS_2_-60-vesicle.

Varying amounts of FeS_2_ particles are introduced into the vesicle solution to investigate the effect of FeS_2_ particle content on the vesicles. As the FeS_2_ content increases, the appearance of the FeS_2_-vesicle solution (Figure 4a,b) varies from clear to turbid gradually. With the same content, the appearance of the vesicle solution with FeS_2_-1 is more turbid than that of the one with FeS_2_-60. Transmittance results (Figure 4c) show that the transmittance of the FeS_2_-vesicle solution decreases with the increase in the content of FeS_2_ particles, whereas there is no change in the transmittance of FeS_2_-water under the same conditions, which is ~99.9%. This excludes the possibility of residual FeS_2_ particles in the FeS_2_-vesicle solution. With the same content, FeS_2_-1 reduces the transmittance more strongly than FeS_2_-60, which is consistent with the variation in appearance (Figure 4a,b). In addition, the decrease in the transmittance of the FeS_2_-1-vesicle solution is 1.6–3.7 times that of the FeS_2_-60-vesicle solution over a range of contents. This can be attributed to the fact that the specific surface area of FeS_2_-1 is larger than that of FeS_2_-60, and the interfacial adsorption and enrichment are more effective.

**Figure 5:**
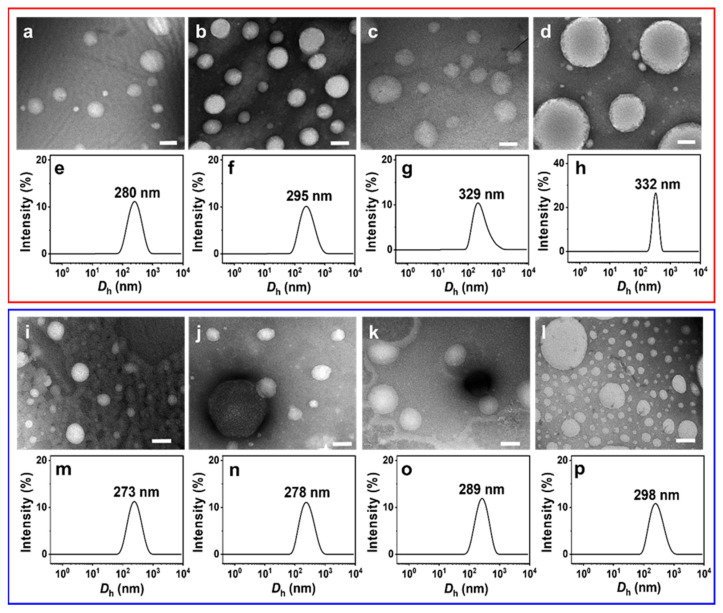
(**a**–**d**,**i**,**j**) NS-TEM images and (**e**–**h**,**m**–**p**) DLS size distributions of (**a**–**h**) FeS_2_-1-vesicle and (**i**–**p**) FeS_2_-60-vesicle solutions at different particle contents (24 h): (**a**,**e**,**i**,**m**) 0.5 g/L; (**b**,**f**,**j**,**n**) 1 g/L; (**c**,**g**,**k**,**o**) 5 g/L; (**d**,**h**,**l**,**p**) 10 g/L. Scale bar: 200 nm.

**Figure 6:**
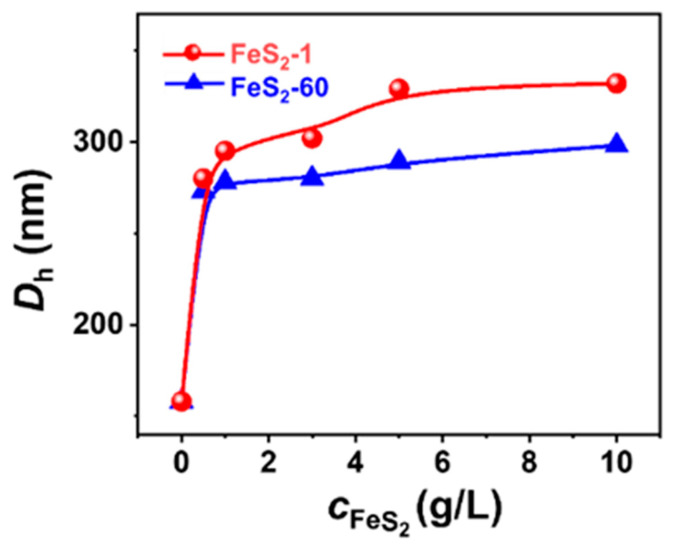
Variation in the DLS size distribution of FeS_2_-vesicle solutions as a function of FeS_2_ content.

NS-TEM results show that the morphology of these vesicles does not change, but the size of the IPN@SDP vesicles grows gradually with the increase in FeS_2_ particle content (Figure 5a–d,i–l). The DLS results indicate that the size of the vesicles in FeS_2_-1-vesicle solution increases from ~158 nm to ~332 nm, while that in the FeS_2_-60-vesicle solution increases to ~298 nm gradually (Figure 5e–h,m–p). This is consistent with the TEM results. As shown in Figure 6, the trend of FeS_2_-1 in inducing a vesicle size increase is greater than that of FeS_2_-60 particles, which is consistent with the variation in effects on the turbidity and the appearance (Figure 6).

In short, the total surface area of FeS_2_ particles in the systems also gradually increases upon increasing FeS_2_ content, which might lead to more remarkable bilayer adsorption and enrichment on the interface of FeS_2_ particles; in other words, the matrix effect is enhanced. This may be the main reason why the vesicle size grows gradually with the increase in FeS_2_ content.

### 2.4. Mechanism of FeS_2_ Particle-Vesicle Solution Interaction

**Figure 7:**
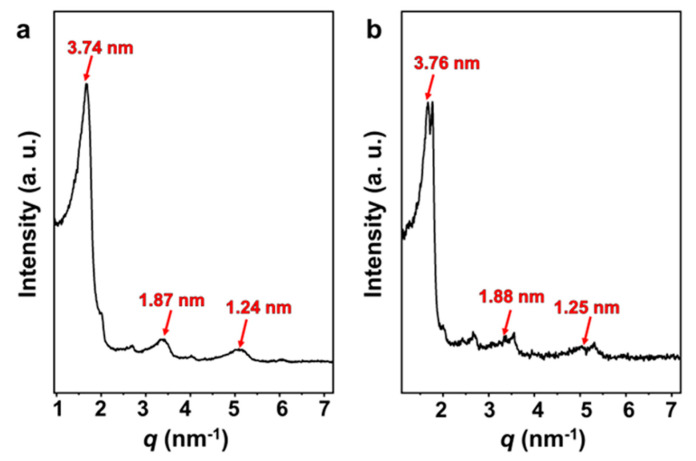
SAXS patterns of (**a**) FeS_2_-1-vesicle and (**b**) FeS_2_-60-vesicle samples.

Optimized by Gaussian generalization theory, the length of the SDP molecule is 1.92 nm [46]. The small-angle X-ray scattering (SAXS) curves (Figure 7) of both the vesicle solution and FeS_2_-vesicle solution samples show lamellar periodic diffraction peaks, indicating the presence of a vesicular bilayer structure. The thickness of the vesicle bilayer is about 3.70 nm (Figure S4), which is less than twice the length of the SDP molecule. This reveals that an interdigitated structure is adopted between the alkyl chains in the vesicular bilayers [46,47,48], with an interdigitated degree of 4.4% (Figure S4). The thicknesses of the vesicle bilayers shift to 3.74 nm and 3.76 nm through the interaction with FeS_2_-1 (Figure 7a) and FeS_2_-60 (Figure 7b), with interdigitated degrees of 3.1% and 2.5%, respectively. In fact, the thickness and interdigitated degrees of the vesicle membranes vary very slightly via the interaction with FeS_2_ particles.

**Figure 8:**
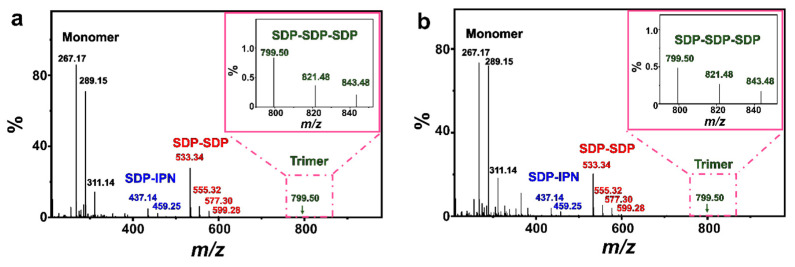
ESI-MS spectra of (**a**) FeS_2_-1-vesicle and (**b**) FeS_2_-60-vesicle samples (24 h). The insets in (**a**,**b**) are magnifications of the square part of the pink dashed line.

Some simple single-chained amphiphilic molecules (SCAs) such as fatty acids [49,50], dodecylhydrogen sulfate [51] and monoalkyl phosphates [22,52,53] can form vesicles spontaneously in water close to their apparent p*K*_a_. The structures of those SCA hydrogen bonding dimers play an important role in this vesicle formation process. As shown by the electrospray ionization mass spectrometry (ESI-MS) results (Figure 8), both FeS_2_-1 and FeS_2_-60 interact with vesicles and show peaks at *m*/*z* of ~289.15 (C_12_H_25_OP(OH)O_2_Na), ~459.25 (C_5_H_9_OH···C_12_H_25_OPO_3_^2−^Na^+^···C_5_H_9_OH) and ~577.30 (C_12_H_25_OP(OH)O_2_Na···C_12_H_25_OP(OH)O_2_Na). This indicates the presence of an SDP monomer, SDP-SDP dimer and IPN-SDP-IPN trimer (Table S1). Compared to the ESI-MS results of the vesicle solution (Figure S5), it is worth mentioning that the FeS_2_-vesicle solution exhibits a new peak at *m*/*z* of 799.50, which indicates the generation of a new hydrogen-bonded trimer (SDP-SDP-SDP). It has been reported that minerals with prebiotic availability may have facilitated prebiotic chemistry by protecting organic molecules from UV radiation and thermal decomposition, concentrating them through adsorption and, finally, catalyzing polymerization reactions [3,54,55]. In this context, the presence of an SDP trimer reveals that pyrite mineral particles have the potential to induce the generation of new substances in simulated primitive Earth environments, which provides new possibilities for exploring the origin of life at the mineral–water interface.

**Figure 9:**
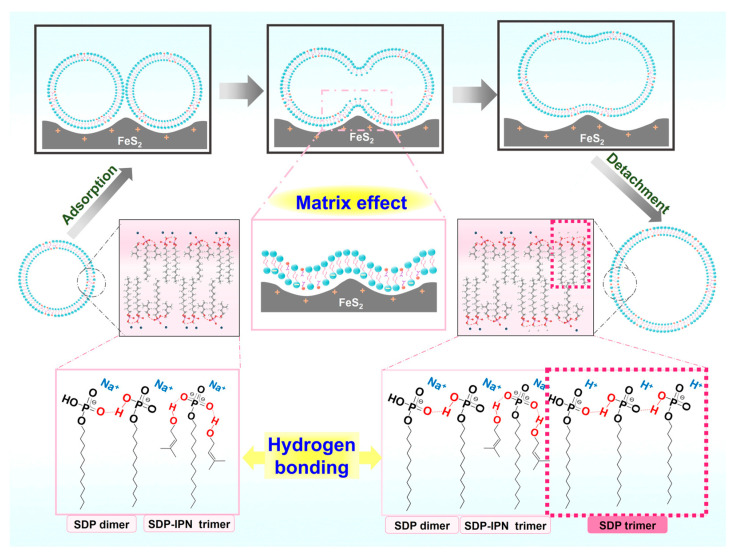
Schematic diagram of FeS_2_-vesicle solution interaction mechanism.

The self-assembly morphology of amphiphilic molecules is determined by their geometrical parameters, which are usually described by the molecular stacking parameter (*P*) defined as *P* = *v*_0_/*a*_s_ *l*_0_ [56,57], where *v*_0_ is the volume of the hydrophobic chain of the amphiphilic molecule, *l*_0_ is the length of the hydrophobic chain, and *a*_s_ is the area occupied by the polar headgroups on the surface of the aggregate. In general, spherical micelles are favored when *P* ≤ 1/3, columnar micelles are favored when 1/3 ≤ *P* ≤ 1/2 and bilayers or vesicles are favored only when 1/2 ≤ *P* ≤ 1. Hydration and electrostatic repulsion between the headgroups can lead to large *a*_s_ values of amphiphiles, whereas the strong adsorption of amphiphiles on solid surfaces through electrostatic interactions, hydrogen bonding and van der Waals forces can significantly reduce the hydration of the headgroups, impede electrostatic repulsion between the headgroups and reduce the *a*_s_ values in amphiphilic molecular aggregates [12,58]. Based on the above background, we propose a possible mechanism of interaction of FeS_2_ particles affecting the vesicle structure (Figure 9). The SDP headgroups are negatively charged, while the FeS_2_ particles are positively charged. The electrostatic interactions allow the SDP monomers and bilayers of SDP/IPN vesicles to be adsorbed onto the surface of these FeS_2_ particles, which reduces the interfacial energy of liquid–solid. This electrostatic adsorption behavior brings about two principle impacts: the fusion of bilayer membranes between vesicles and the growth of vesicles.

(1) *Fusion of bilayer membranes between vesicles*. The electrostatic adsorption and the matrix effect [43] of the FeS_2_–water interface increase the probability of contact between the vesicular bilayers. The “fast exchange” and “flip-flop” characteristics [59] of vesicles enable the contact or near enough area of the bilayer membranes to fuse with each other.

(2) *Growth of vesicles*. The FeS_2_–water interface induces the dehydration of the SDP polar headgroups significantly and impedes electrostatic repulsion between the headgroups. It results in the diminution of the cross-sectional area (*a*_s_) occupied by the SDP polar headgroups on the surface of the vesicle structures [12,58]. The reduced *a*_s_ decreases the curvature of the bilayer membranes, which leads to a lager radius of the vesicles. Cooperating with the fusion between vesicular bilayers, it results in the fusion–growth of vesicles.

As this process is repeated, the vesicles fuse and grow gradually until they reach the equilibrium of their transition (sphere–dumbbell–ellipsoid–larger sphere vesicles) (Figure 5). During this process, the FeS_2_–water interface drives the formation of brand new hydrogen-bonded trimers (SDP-SDP-SDP) (Figure 8). Furthermore, the dumbbell-shaped and elliptical vesicle structures observed during this process (Figure 3) also corroborate this speculation.

In conclusion, based on the results of DLS, NS-TEM and ESI-MS (Figure 3, Figure 5 and Figure 8), the electrostatic adsorption between positively charged FeS_2_ particles and negatively charged SDP molecules drives the vesicles to be adsorbed and enriched on the interface of FeS_2_ particles, which further fuse and grow into vesicles with larger sizes. The vesicle fusion–growth process is influenced simultaneously by the electrostatic adsorption, matrix effects and the dehydration effects of the solid interface.

## 3. Materials and Methods

### 3.1. Materials

All chemicals used were of analytical reagent grade and utilized as received. Sodium monododecyl phosphate (SDP) was procured from TCI (Shanghai) Development Co., Ltd., Shanghai, China. Isopentenol (IPN) and natural pyrite (FeS_2_) were sourced from Macklin Biochemical Technology Co., Ltd., Shanghai, China. Ultrapure water was obtained using a Hitech-Kflow water purification system (Hitech, Shanghai, China).

### 3.2. Size Regulation of FeS_2_ Particles

Natural pyrite was ground into a granular form using an agate mortar and pestle. Pyrite particles with sizes ranging between the 200-mesh screen and the 290-mesh screen, corresponding to a size range of 60 ± 15 μm, were selected and designated as FeS_2_-60. Additionally, pyrite was ground into a powder form, sieved through a 2800-mesh screen and characterized using a nanoparticle sizer and an optical microscope, revealing a size of approximately 1 μm, and it was recorded as FeS_2_-1.

### 3.3. Preparation of Vesicle Solutions

Accurately weighed SDP was mixed with a designated amount of water, and a desired mass of IPN was added to the SDP/H_2_O mixture. The sample was homogenized by shaking and vortexing and then kept at 25.0 ± 0.5 °C for 48 h to achieve equilibrium before measurements. The composition of the SDP/IPN/H_2_O vesicular solution was fixed to be 0.30 wt% SDP (~10 mM) and 5.00 wt% IPN hereinafter if not otherwise specified.

### 3.4. Preparation of Particle–Solution Complexes

The FeS_2_ particle was introduced into the SDP/IPN/H_2_O solution and subjected to mixing by shaking and vortexing. The supernatant was investigated through centrifugation at 2000 rpm for 3 min using a high-speed centrifuge. If not otherwise indicated, FeS_2_ was incorporated into the solution at a content of 3 g/L, and the vesicle solution was sampled with a composition of 0.30 wt% SDP and 5.00 wt% IPN. The resultant supernatant after interaction with the FeS_2_ particle was noted as the FeS_2_-vesicle solution. The FeS_2_-vesicle solutions were kept at 25.0 ± 0.5 °C for at least 24 h after the addition of FeS_2_ particles for the experiments in Section 2.3.

### 3.5. Characterization and Measurements

#### 3.5.1. Scanning Electron Microscopy (SEM)

SEM images of samples were acquired using a Gemini 300 field-emission scanning electron microscope (Zeiss, Oberkochen, Germany) operated at an accelerating voltage of 3 kV. The sample solution was freeze-dried in a vacuum freeze dryer at approximately −40°. Subsequently, the freeze-dried samples were mounted on a 200-mesh C-coated grid and coated with a 5 nm thick layer of Pt using sputter coating.

#### 3.5.2. Optical Microscope (OM)

An XPF-800C optical microscope (Tianxing, Shanghai, China) was employed to examine the morphology of the samples. Subsequently, the acquired images were processed using Nano Measurer 1.2 software to conduct size analysis and generate a histogram depicting the sample size distribution.

#### 3.5.3. Dynamic Light Scattering (DLS) Measurements

The size distribution and average hydrodynamic diameter (*D*_h_) of the aggregates were assessed using a Zetasizer Nano ZS90 dynamic light scattering instrument (Malvern, Worceterhire, UK) featuring a He-Ne laser (633 nm, 4 mW). Each sample underwent three measurements at 25.0 ± 0.5 °C.

#### 3.5.4. Zeta Potential Measurement

A ZetaNano ZS-type zeta potential analyzer (Malvern, Worceterhire, UK) was employed to investigate the variation in zeta potential with pH in FeS_2_ aqueous suspensions. The pH of the FeS_2_ particle–water suspension was adjusted using diluted hydrochloric acid or sodium hydroxide, with the FeS_2_ content set at 3 g/L. Temperature control achieved using a thermostatic water bath maintained at 25.0 ± 0.5 °C. Measurements were conducted three times concurrently, and the average values were calculated.

#### 3.5.5. pH Measurement

A pH meter (Mettler Toledo, Shanghai, China) was utilized to measure the variation in pH of the sample solution. Measurements were performed three times concurrently, and the average value was calculated.

#### 3.5.6. Contact Angle Meter

The water contact angle (*θ*_w_) of the samples was determined by the sitting drop method using a DSA25 contact angle meter (KRÜSS, Hamburg, Germany). Firstly, the powder was pressed into a disc with a thickness of 1–2 mm under a pressure of 15 MPa, and then placed on the sample stage. The computer controlled the speed and volume of the droplets of the micro-syringe, and the droplets were dropped after stabilization, and the values of the droplet contact angle were photographed and recorded after equilibrium for 10 s. Each sample was tested three times, and the average value of the three tests was taken.

The FeS_2_ particle–vesicle solution complexes were centrifuged at 2000 rpm for 3 min to remove the supernatant at 25.0 ± 0.5 °C. FeS_2_ particles were washed 3 times by adding ultrapure water, and then air dried and recorded as FeS_2_ mixed with vesicle solution. The dried FeS_2_ samples were subjected to ultrasonic treatment in ultrapure water for 5 min, and then were centrifuged and air dried, noted as FeS_2_ with ultrasonic treatment. The water contact angles of FeS_2_ particles, FeS_2_ mixed with vesicle solution and FeS_2_ with ultrasonic treatment were measured separately.

#### 3.5.7. Specific Surface Area and Pore Size Analyzer

The specific surface area and pore size distribution were obtained using an ASAP2460 specific surface area and pore size analyzer (Micromeritics, Norcross, USA) to determine the nitrogen adsorption–desorption isotherms of the samples under the condition of liquid nitrogen, and the specific surface area and pore size distribution were determined by using the BET method and BJH model. The samples were degassed under vacuum and 100 °C for 3 h before testing.

#### 3.5.8. Ultraviolet Spectrophotometer (UV-Vis)

The transmittance of the samples was measured at a wavelength of 500 nm using a model 1800 UV-Vis spectrophotometer (Shimadzu, Kyoto, Japan) maintained at a constant temperature of 25.0 ± 0.5 °C. Quartz cuvettes with plastic caps were employed to prevent sample evaporation during analysis. Each liquid sample was placed in a quartz cuvette with a plastic lid to ensure sample integrity. Measurements were conducted three times concurrently, and the average value was calculated.

#### 3.5.9. Negative Staining Transmission Electron Microscopy (NS-TEM)

The morphology of the aggregates was observed using uranyl acetate negative staining on a JEM-1011 transmission electron microscope (JEOL, Tokyo, Japan) operated at an accelerating voltage of 100 kV. A 10 μL aliquot of the sample was applied onto a carbon support film copper grid with a mesh size of 200 and allowed to settle for 2 min. Excess sample was blotted away using filter paper, followed by the addition of 7.5 μL of a 1.2% uranyl acetate ethanol solution. After 30 s, excess solution was removed by blotting with filter paper. Subsequently, the copper grid was dried under an infrared lamp for 30 min before the sample was transferred to a desiccator for electron microscope observation.

#### 3.5.10. Small-Angle X-ray Scattering (SAXS)

The SAXS patterns were obtained using a SAXSess system (Anton-Paar, Graz, Austria) equipped with Cu Kα radiation and operated at 50 kV and 40 mA. Prior to measurements, the samples underwent freeze-drying under vacuum conditions.

#### 3.5.11. Electrospray Ionization Mass Spectrometry (ESI-MS)

Mass spectrometry data were acquired in positive ion mode using a Bruker Impact M1 ultra-high performance liquid chromatography–quadrupole time-of-flight mass spectrometer (Bruker, Karlsruhe, Germany).

#### 3.5.12. Molecular Dynamics Simulation

The molecular modeling and theoretical investigation for SDP dimers and trimers were carried out with LDA-DFT as implemented in the Dmol3 package provided by Materials Studio 2019. The local functional for the exchange correlation potential is LDA-PWC. The basis set is DND with unrestricted spin, minimum basis set, 3.5 basis files and fine cut-off [60]. The convergence tolerances for energy change, maximum force and maximum displacement between optimization cycles were set as 1.0 × 10^−5^ Ha, 0.002 Ha Å^−1^ and 0.005 Å, respectively [61].

## 4. Conclusions

In summary, we selected primitive relevant compounds (SDP, IPN and FeS_2_) to construct a protocell model in water. The addition of FeS_2_ particles enables the fusion and growth of IPN@SDP vesicles, induces the morphology transition of sphere–dumbbell–ellipsoid–larger sphere vesicles and reaches the kinetic equilibrium in ~12 h. The mediating behavior of pyrite solid–water interfaces increases the vesicle size from ~150 nm to ~300 nm, and the transmittance decreases gradually from ~99% to 94–83%. In particular, the pyrite solid–liquid interfacial mediating capacity of pyrite particles with small particle sizes (~1 μm, FeS_2_-1) is more significant than the larger one (~60 μm, FeS_2_-60), which may be due to the larger specific surface area of FeS_2_-1. As the FeS_2_ particle content increases, the total surface area increases, and the more pronounced the matrix effect at the solid–liquid interface is, inducing an increase in vesicle size. Briefly speaking, the matrix effect, intermolecular hydrogen bonding, and electrostatic interaction is demonstrated to be the main driving force for the fusion–growth behavior of the vesicle, the process in which the FeS_2_–water interface drives the formation of the new substance (hydrogen-bonded trimers). This opens up the possibility for the generation of new substances in the prebiotic solid–liquid interface scenario. We expect this work to provide important insights into the effect of the solid–liquid interface on the self-assembly chemistry of SCAs and OSMs with primitive relevance in bulk solution, which, in the long run, may shed some light on the establishment of the model systems of early cell membranes for exploring the origin of life.

## Data Availability

The data presented in this study are available on request from the corresponding author.

## Supplementary Materials

The following supporting information can be downloaded at https://www.mdpi.com/article/10.3390/molecules29112664/s1: Figure S1: Vesicle phase diagram of the SDP/IPN/H_2_O ternary system at 25.0 ± 0.5 °C; Figure S2: The water contact angles on the surface of (a,b) FeS_2_ mixed with vesicle solution and (c,d) FeS_2_ with ultrasonic treatment. (a,c) FeS_2_-1, (b,d) FeS_2_-60; Figure S3: (a,d) Photographs, (b,e) NS-TEM images and (c,f) DLS size distribution in (a–c) FeS_2_-1-vesicle and (d–f) FeS_2_-60-vesicle at 25.0 ± 0.5 °C over six months. Scale bar: 200 nm; Figure S4: SAXS patterns of vesicle samples; Table S1: Chemical structures corresponding to different *m*/*z* in ESI-MS spectra; Figure S5: ESI-MS spectra of vesicle samples (24 h).
