# Supplementary material for: A Fusion–Growth Protocell Model Based on Vesicle Interactions with Pyrite Particles
Source: Molecules. 2024 Jun 4;29(11):2664. doi: 10.3390/molecules29112664 (PMC11173516; doi:10.3390/molecules29112664)
Supplement: Supplementary file 1 [file molecules-29-02664-s001.zip › molecules-3006342-supplementary.pdf]

# Supporting Information (SI)

# A Fusion–Growth Protocell Model Based on Vesicle Interactions with Pyrite Particles

**Dong Guo [1], Ziyue Zhang [1], Jichao Sun [1], Hui Zhao [2], Wanguo Hou [1,2] and Na Du [1,*]**

[1] Key Laboratory of Colloid and Interface Chemistry (Ministry of Education), School of Chemistry and Chemical Engineering, Shandong University, Jinan 250100, China
[2] National Engineering Technology Research Center for Colloidal Materials, Shandong University, Jinan 250100, China
[*] Correspondence: duna@sdu.edu.cn; Tel.: +86-531-88364242

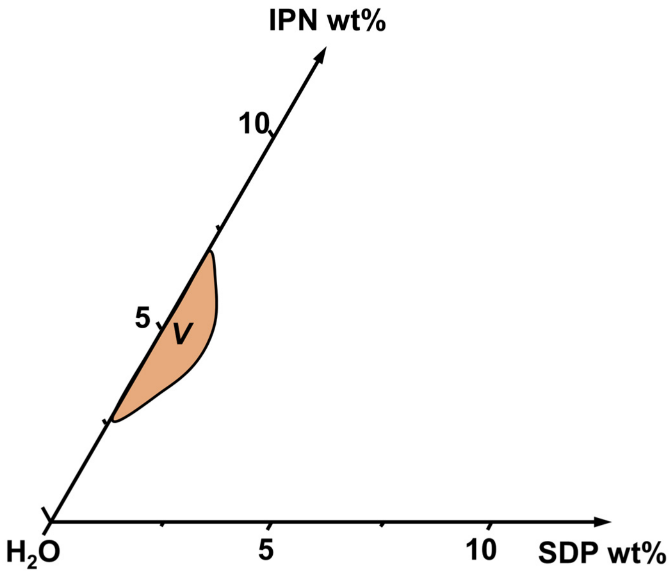


**Figure S1.** Vesicle phase diagram of the SDP/IPN/$H_2O$ ternary system at 25.0 ± 0.5 °C.

Phase diagrams are established by preparing samples over varying mass percentages of SDP (from 0.00 to 80.00 wt%). Firstly, the required amounts of SDP and water were added into well-sealed glass vials to obtain a series of suspensions. Thereafter, a trace amount of IPN was introduced into each suspension by the successive addition method. After being homogenized by vibration and vortex, the obtained samples were placed in a thermostatic bath (25.0 ± 0.5 °C) for a few hours to days to attain equilibrium. To identify the limits of the different phases in the diagram, the macroscopic appearances (clarity/turbidity and homogeneous/heterogeneous) of the samples and the morphologies and sizes of the aggregates in the samples were characterized by visual observation, transmission electron microscopy (TEM), and dynamic light scattering (DLS) measurements. The orange area in **Figure S1** represents the vesicle phase.

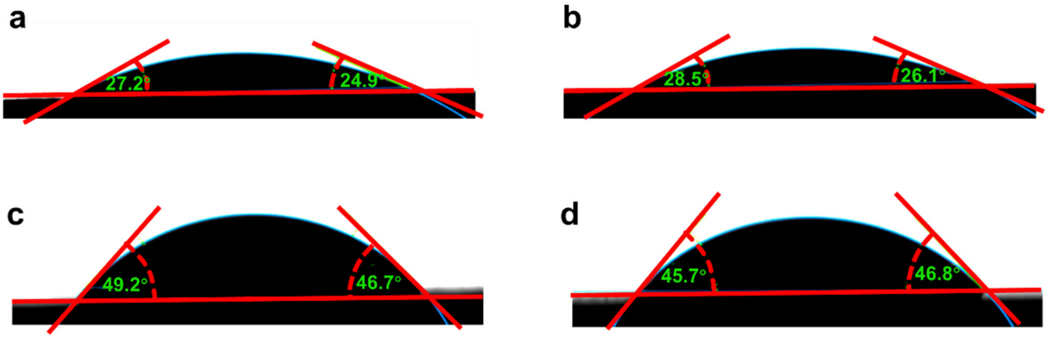


**Figure S2.** The water contact angles on the surface of (**a**, **b**) $FeS_2$ mixed with vesicle solution and (**c**, **d**) $FeS_2$ with ultrasonic treatment. (**a**, **c**) $FeS_2$−1, (**b**, **d**) $FeS_2$−60.

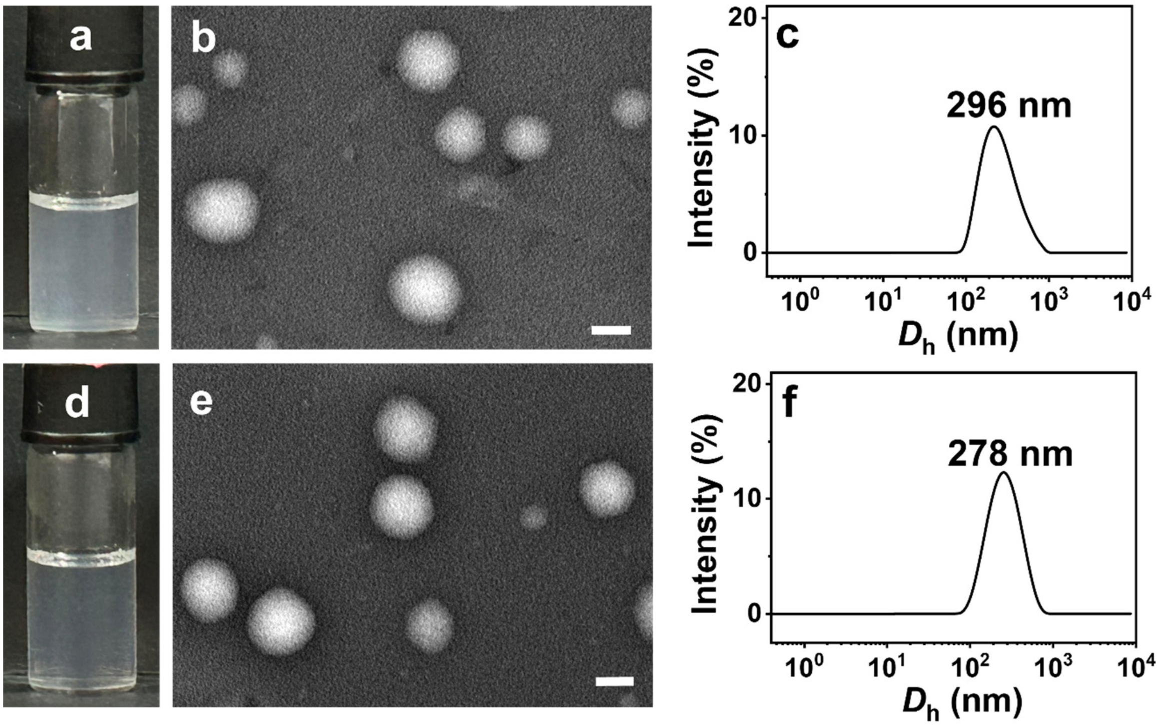


**Figure S3.** (**a**, **d**) Photographs, (**b**, **e**) NS-TEM images, and (**c**, **f**) DLS size distribution in (**a**-**c**) $FeS_2$−1−vesicle and (**d-f**) $FeS_2$−60−vesicle at 25.0 ± 0.5 °C over six months. Scale bar: 200 nm.

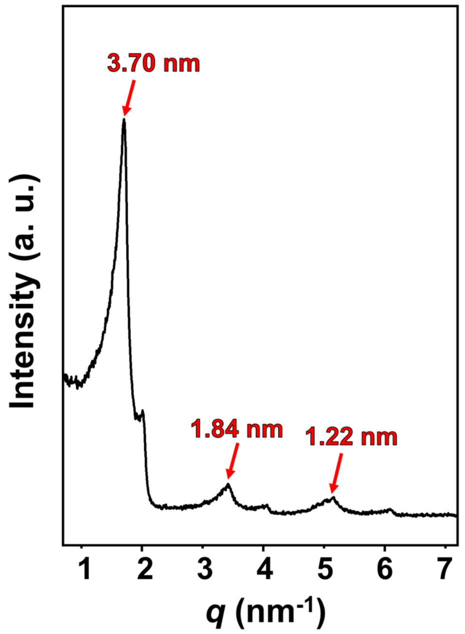


**Figure S4.** SAXS patterns of vesicle samples.

**Table S1.** Chemical structures corresponding to different *m/z* in ESI-MS spectra.

| *m/z* | chemical structure |
|---|---|
| 267.17 | $C_{12}H_{25}O_2P(OH)_2$ |
| 289.15 | $C_{12}H_{25}OP(OH)O_2Na$ |
| 311.14 | $C_{12}H_{25}OPO_3Na_2$ |
| 437.14 | $C_5H_9OH\cdots C_{12}H_{25}OPO_3^{2-}\cdots C_5H_9OH$ |
| 459.25 | $C_5H_9OH\cdots C_{12}H_{25}OPO_3^{2-}Na^+\cdots C_5H_9OH$ |
| 533.24 | $C_{12}H_{25}O_2P(OH)_2\cdots C_{12}H_{25}O_2P(OH)_2$ |
| 555.32 | $C_{12}H_{25}O_2P(OH)_2\cdots C_{12}H_{25}OP(OH)O_2Na$ |
| 577.30 | $C_{12}H_{25}OP(OH)O_2Na\cdots C_{12}H_{25}OP(OH)O_2Na$ |
| 599.28 | $C_{12}H_{25}OP(OH)O_2Na\cdots C_{12}H_{25}OPO_3Na_2$ |
| 799.50 | $C_{12}H_{25}O_2P(OH)_2\cdots C_{12}H_{25}O_2P(OH)_2\cdots C_{12}H_{25}O_2P(OH)_2$ |
| 821.48 | $C_{12}H_{25}O_2P(OH)_2\cdots C_{12}H_{25}OP(OH)O_2Na\cdots C_{12}H_{25}O_2P(OH)_2$ |
| 843.48 | $C_{12}H_{25}OP(OH)O_2Na\cdots C_{12}H_{25}O_2P(OH)_2\cdots C_{12}H_{25}OP(OH)O_2Na$ |

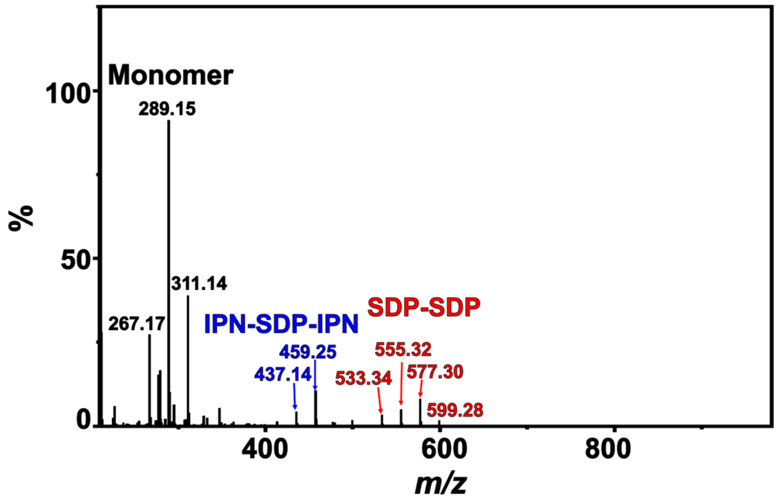


**Figure S5.** ESI-MS spectra of vesicle samples (24 h).
